# The effect of taping versus semi-rigid bracing on patient outcome and satisfaction in ankle sprains: a prospective, randomized controlled trial

**DOI:** 10.1186/1471-2474-13-81

**Published:** 2012-05-28

**Authors:** Sacha Lardenoye, Ed Theunissen, Berry Cleffken, Peter RG Brink, Rob A de Bie, Martijn Poeze

**Affiliations:** 1 Department of Surgery, Division of Traumasurgery, Maastricht University Medical Center+, P Debyelaan 25, 6202 AZ, Maastricht, The Netherlands; 2 Department of Epidemiology, Maastricht University, P.O. Box 616, 6200 MD, Maastricht, The Netherlands; 3 Department of Surgery, Maasstad Ziekenhuis, Maasstadweg21, 3079 DZ, Rotterdam, The Netherlands

## Abstract

**Background:**

Functional treatment is a widely used and generally accepted treatment for ankle sprain. A meta-analysis comparing the different functional treatment options could not make definitive conclusions regarding the effectiveness, and until now, little was known about patient satisfaction in relation to the outcome.

**Methods:**

Patients with acute ankle sprain received rest, ice, compression and elevation with an compressive bandage at the emergency department. After 5-7 days, 100 patients with grade II and III sprains were randomized into two groups: one group was treated with tape and the other with a semi-rigid ankle brace, both for 4 weeks. Post-injury physical and proprioceptive training was standardized. As primary outcome parameter patient satisfaction and skin complications were evaluated using a predefined questionnaire and numeric rating scale. As secondary outcome parameter the ankle joint function was assessed using the Karlsson scoring scale and range of motion.

**Results:**

Patient-reported comfort and satisfaction during treatment with a semi-rigid brace was significantly increased. The rate of skin complication in this group was significantly lower compared to the tape group (14.6% versus 59.1%, P < 0.0001). Functional outcome of the ankle joint was similar between the two treatment groups, as well as reported pain.

**Conclusion:**

Treatment of acute ankle sprain with semi-rigid brace leads to significantly higher patient comfort and satisfaction, both with similar good outcome.

## Background

Acute ankle sprain is one of the most common musculoskeletal injuries, accounting for an estimated 600.000 persons per year in the Netherlands [1]. Fifty percent of these injuries arise in sports and in seventy-five percent the cause is an inversion trauma [1]. Recent research showed that, in the Netherlands, the mean total cost (direct and indirect) of one ankle sprain was about €360 [2] giving an annual cost of approximately €100 million in the Netherlands alone. In the United States ankle sprains occur in an estimated 23.000 people per day which equals about 8.4 million people per year [3].

Functional treatment (meaning non-operative and non-immobilizing therapy) is a commonly accepted treatment of ankle sprains. Functional treatment includes a wide variety of options. The most common functional treatment methods used in the Netherlands are taping or bracing which have superior functional results compared to plaster immobilization and elastic bandage [4,5]. A meta-analysis comparing the different functional treatment options (which included elastic bandage, tape, semi rigid ankle support, and lace up ankle support) could not make definitive conclusions, because diversity of outcome results prohibited pooling of different studies [5-7]. In addition, tape treatment resulted in significantly more complications, the majority being skin irritations, when compared with treatment with an elastic bandage [5,8]. Recent reviews indicated identical conclusions regarding functional treatment of ankle sprains [9,10].

Therefore, several questions remain to be answered with regard to the patient satisfaction during the functional treatment of acute ankle sprain. The hypothesis tested in this study is that the treatment of lateral ankle sprain with a semi-rigid brace leads to less local complications [allergic contact dermatitis, bullae, and skin pressure abnormalities] and more patient satisfaction than treatment with tape. Reduction in complications will improve patient satisfaction with the treatment method and this will improve functional outcome by enhancing compliance with the treatment method used. The aim of this study was to determine the effect of treatment with tape compared to treatment with brace on patient outcome and satisfaction in ankle sprains.

## Methods

### Study design

Prospective, randomized controlled trial conducted in a trauma out patient clinic from February 2008 till July 2009. The Institutional Review Board of the Maastricht University Medical Center (MEC072094/NL20031.068.07) approved this study. Written informed consent was obtained from each patient.

### Inclusion/exclusion criteria

Patients were included if they sustained a grade II or III ankle sprain (significant damage to lateral ligaments defined by the presence of a lateral hematoma and tenderness at the anterior lateral ligament without (grade II) or with anterior drawer instability (grade III) as assessed by a supervised resident or by the treating physician when presented in the outpatient clinic within 5-7 days. Grade I ankle sprain was determined as the absence of a hematoma and tenderness at the anterior lateral ligament. Patients with the presence of a lateral hematoma and tenderness at the anterior lateral ligament without instability were defined as grade II) as patient with lateral hematoma, tenderness and instability were defined as grade III [3]. The study excluded patients undergoing preventive treatment of recurrent ankle sprains. The specificity and sensitivity of delayed physical examination for the presence of absence of a lateral ankle ligament rupture are 84% and 96%, respectively. A positive anterior drawer test in combination with pain on palpation on the ATFL and hematoma discoloration has a sensitivity of 100% and specificity of 77% [11]. Patients were excluded if they had a fracture, if their age was under 16 or over 55 years; if they had experienced a previous ankle sprain or fracture; if they sustained swelling that made treatment with tape impossible, were mentally disabled or were unwilling to participate in the study.

### Randomization and treatment

Patients with an inversion trauma were physically examined by a physician assistant or junior resident at the emergency department. A fracture was excluded, following the Ottawa Ankle Rules [12,13]. In case of a sprain, initial treatment consisted of a compressive bandage together with a standard advice (rest, ice, compression, and elevation). Pain medication and crutches were not standardized. Patients revisited the outpatient clinic within 5-7 days after the trauma. At that time, a supervised resident or treating physician reassessed the ankle. After informed consent, patients with a grade II/III lateral ankle sprain were randomized into two equal groups. An independent research assistant performed a concealed permuted block randomization using a computer-generated randomization schedule with a random block size. Treating physicians and patients were blinded to the randomization process. One group was treated with tape (Coumans-bandage) and the other with a semi-rigid brace (AirLoc ® Bauerfeind, Zeulenroda, Germany), both for 4 weeks. The tape was re-applied in the outpatient clinic at least once after two weeks or when patients indicated that stability was lost from the tape or for hygiene purposes or skin related problems. Taping was performed by a select group of experienced and skilled healthcare professionals of the outpatient clinic. The tape consists of three layers. The first layer is a latex free, adhesive, bandage to protect the skin. The second layer consists of 2.5 cm non-elastic strapping tape (Leukotape, Beiersdorff) used for support. The third layer consists of elastoplasts 6 cm broad, elastic used for fixation of the second layer [14].The ankle semi-rigid ankle brace used has contoured plastic shells that are held in place with hook and loop fasteners that can be adjusted individually. This ankle support (medial and lateral side of the ankle) has air cushions that inflate to stabilize the ankle’s lateral ligaments preventing them from twisting.

Supervised proprioceptive exercises were given in both groups, starting one week after trauma. Verbal and written instructions for daily home exercises, focused on proprioceptive, range of motion training and strength exercises, were given by the attending nurse. During follow up additional instructions could be given. Follow up took place at week 3, 5, 9 and 13 post injury which was indicated in the study as week 2, 4, 8, and 12 after start of the study treatment.

### Outcome

As primary outcome parameter patient satisfaction was assessed by verbal rating scale: poor (5), moderate (4), sufficient (3), good (2) and excellent (1) both at 2 and 4 weeks after start of the study treatment. In addition, the ankle joint function was assessed using the validated Karlsson scoring scale [15] and range of motion at 2, 4, 8 and 12 weeks after start of the study treatment. An anterior drawer test was used to assess the stability of the anterior talofibular ligament and compared to the uninjured ankle. The Karlsson scoring scale (Table 1) consists of eight categories with a total of 90 points, assessing pain, swelling, instability, stiffness, stair climbing, running, work activities and support. Also, the level of pain was evaluated using a 5 point pain scale: no pain (1) mild pain (2) moderate pain (3) severe pain (4) overwhelming pain/worst ever (5). The same 5-point Likert scale was used to assess patient-reported hygiene. Complications of the treatment were registered as allergic contact dermatitis, bullae and/or skin pressure abnormalities requiring local skin treatment or cessation of the treatment.

**Table 1:** Karlsson scoring scale

**Category**	**degree**	**Score**
Pain	None	20
	During exercise	15
	Walking on uneven surface	10
	Walking on even surface	5
	Constant	0
Swelling	None	10
	After exercise	5
	Constant	0
Instability (giving way)	None	15
	Walking on uneven ground	10
	Walking on even ground	5
	Constant using support	0
Stiffness	None	5
	Moderate (morning, exercise)	2
	Constant	0
Stair Climbing	No problems	10
	Impaired	5
	Impossible	0
Running	No problems	10
	Impaired	5
	impossible	0
Work activities	Same as before injury	15
	Same work, less sports	10
	Ligther work, no sports	5
	Severely impaired work, decreased leisure activities	0
Support	None	5
	Ankle support during exercise	2
	Ankle support during daily activities	0
Total		90

The range of motion of the ankle joint covers the movement between maximum dorsal and maximum plantar flexion. The foot was placed in the neutral position, using the Neutral-0-method. The goniometer was aligned along metatarsal I with the border of the instrument just proximal to the head of the metatarsal to ensure comparable placement at each visit, then ROM was measured using an electronic goniometer (Hoggan Health Ind, West Jordan, UT, USA).

### Sample size

The minimum sample size is calculated for 90% power of testing and a 5% level of significance (α = 0.05, β = 0.10) a minimum of 36 patients per group is required for this study. With a 20% drop-out and lost-to-follow up rate a minimum total number of 87 patients should be included in the study with complete follow-up to demonstrate an improved patient satisfaction of 15% (baseline values 2.6 (SD 0.5) (from pilot study (data not shown) compared to an expected value of 2.2 (SD 0.5) in the group of 72 patients with complete data. Other parameters used to confirm the adequacy of the sample size was the Karlsson score that was expected to increase from 35 (SD 15) during tape treatment to 50 (SD 20) in the brace group (number needed with complete data to include 2x 24 patients).

### Statistics

Data were analyzed with SPSS version 16.0 (Statistics Package for the Social Sciences, SPSS Inc. Chicago, Illinois 60606). Data from the demographic data collection and the outcome parameters was cleaned blindly from the treatment data. Missing data of individual patients were replaced by the mean of the series of the allocated treatment group. Data were presented as mean scores with 95% confidence intervals. The analysis of this study was performed according to the intention-to-treat principle. Analysis of functional outcome and patient satisfaction was assessed using repeated-measures analysis of variance with correction of the degrees of freedom using the Greenhouse-Geisser estimates of sphericity when Mauchly’s test indicated that the assumption of sphericity had been violated using the parameter time as the within-group factor and treatment as the between-group factor. Group comparisons at the different time points were only performed when the overall repeated-measures tests were statistically significant. Bonferroni correction for multiple testing was performed with adjustment of the multiple testing. Sensitivity analysis for missing data was performed to verify overall repeated-measures outcomes. All scores were tested for normality using the Kolmogorov-Smirnov test. Parametric variables were compared using the Student’s *t*-test, while non-parametric and ordinal variables were compared using the Mann–Whitney U statistic. Nominal variables were compared across independent groups using the chi-squared test or Fisher’s exact test. Homogeneity of variance was assessed using Levene’s test. No post-hoc analyses were performed. Level of significance was set at P < 0.05.

## Results

### General demographics

**Figure 1:**
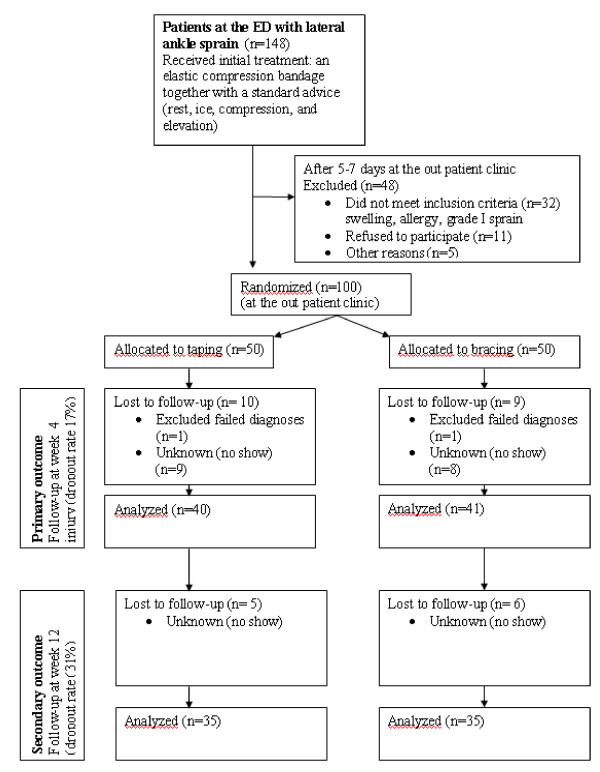
**Flow chart of the randomized trial comparing tape treatment and treatment with a semi-rigid brace.** ED emergency department.

In total 100 patients were included in the study and randomized after initial treatment and screening (Figure 1). Two patients were considered non-eligible after randomization: both patients had a fracture at control X-ray and fulfilled exclusion criteria. The results regarding primary outcome (patient satisfaction, complications and pain) were completed for 81 (83%) patients (nine patients in the tape group and 8 patients in the brace group were lost from follow-up). The results regarding secondary outcome (ankle function) were completed for 70 (71%) patients.

Patients’ age and gender were similar between the two groups. In total 38% of patients sustained the ankle sprain due to sport related activities, which was distributed similarly between the two treatment groups (tape: 19/49 versus 18/49, p = 0.8) (Table 2). The number of positive anterior drawer test of the injured ankle compared to the uninjured ankle was similar prior the start of the treatment (1/49 versus 2/49 in the tape group versus semi-rigid brace group, respectively, p = 0.2).

**Table 2:** Characteristics of patients according to allocated treatment

	**Tape (N = 49)**	**Semi-rigid brace (N = 49)**	**P-value**
No. of females	23	16	0.1
Mean (SD) age (years)	30	29.8	0.9
Percentage sport related injury	39% (19/49)	37% (18/49)	0.8
Percentage grade III ankle sprain	2% (1/49)	4% (2/49)	0.2

### Patient satisfaction and treatment complications

**Figure 2:**
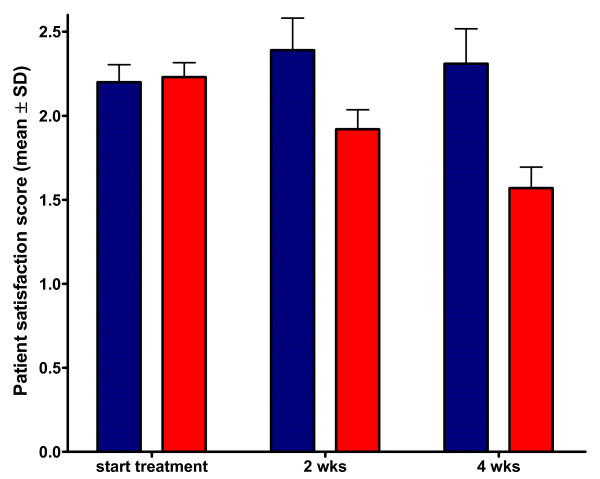
**Patient reported satisfaction during functional treatment of acute ankle sprain.** Data are presented as mean (+SD) on a Likert scale ranging from 5 (poor) to 1 (excellent). Patient satisfaction was significantly better in the patients treated with a semi-rigid brace compared to tape treatment (P < 0.0001).

Repeated-measures analysis of variance revealed there was a significant interaction effect for the parameter satisfaction (Wilks’Lambda 0.9; F 12.9; partial eta squared 0.052; P_TxG_ <0.0001). Posthoc testing revealed that during the 4 week treatment period patient satisfaction was significantly higher in the patient group treatment with a brace at 3 and 5 weeks (P < 0.05; Figure 2). While satisfaction in the tape treatment significantly decreased from week 1 till week 5 (P < 0.05), the patient rated satisfaction improved significantly in the patients treated with a brace comparing week 3 with the start of the treatment.

Of all patients treated with tape 59.1% experienced complications, including contact dermatitis, bullae formation or skin abnormalities due to increased local pressure, requiring local skin treatment or cessation of the treatment. This rate of complications was significantly lower in the brace group (14.6%, P < 0.0001). These results were also reflected by the experienced hygiene during treatment. At all measured time-points the reported hygiene was significantly higher in the patients treated with brace (Group effect: F 5.3; partial eta squared 0.125; P_G_ < 0.0001, Time effect: Wilks’Lambda 0.948; F 5.310; partial eta squared 0.029; P_T_ = 0.02, Interaction: Wilks’Lambda 0.997; F 0.332; partial squared eta 0.045; P_TxG_ = 0.6).

During the trial two (4.1%) crossovers were found from brace to tape, due to less stability reported when using the brace. No crossovers from tape to brace were found (P = 0.1).

### Functional outcome

**Figure 3:**
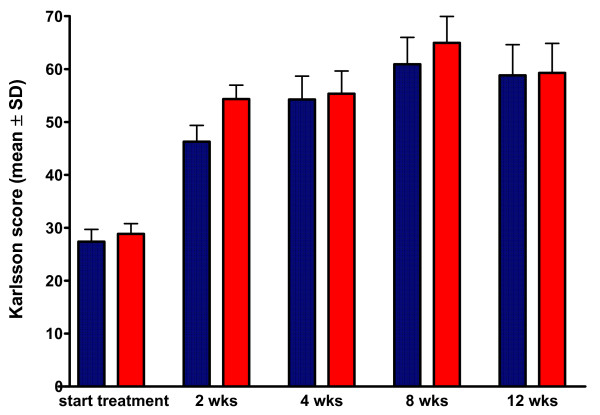
**Karlsson function outcome score during functional treatment of acute ankle sprain.** Data are presented as mean (+SD) on a scale ranging from 0 (poor) to 100 (optimal). Fuctional outcome reported by the patients increased significantly (P_T_ < 0.0001) during the 4 weeks treatment and further increased thereafter until 8 was similar between semi-rigid brace and tape treatment (P = 0.4).

The functional outcome as assessed using the Karlsson score increased significantly (Time effect: Wilks Lambda 0.438; F 29.822; partial eta squared 0.562; P_T_ < 0.0001) during the 4 weeks treatment and further increased thereafter until 8 weeks, after which the functional level stabilized at a mean score of 84 (SD 11) of maximal 90 points (Figure 3). There was no difference in this increased functional ability between the two groups (Group effect: F 0.492; partial eta squared 0.005; P_G_ = 0.5) (Figure 3), including time to return to normal work and sport activities. In addition, the pain score was similar between the tape and brace treatment (Group effect: F 0,277, partial eta squared 0.003; P_G_ = 0.4, Time effect: Wilks’Lambda 0.526; F 18.023; partial eta squared 0.474; P_T_ < 0.0001, Interaction: Wilks’ Lambda 0.924; F 1.651, partial eta squared 0.076; P_TxG_ = 0.4).

The passive and active range of motion, expressed as the difference between the uninjured and injured ankle improved similarly in both the patients treated with a brace and the patients treated with taping (Table 3).

**Table 3:** Active and passive range of motion after functional treatment of acute ankle sprain

	**Treatment**	**N**	**Mean**	**SD**	**P-value**
Passive ROM -week 4	tape	39	12.5	8.9	0.9
	semi-rigid brace	41	12.3	11.3	
Active ROM - week 4	tape	39	13.7	9.0	0.7
	semi-rigid brace	41	12.8	14.1	
Passive ROM - week 12	tape	34	3.6	6.4	0.2
	semi-rigid brace	35	5.8	7.6	
Active ROM - week 12	tape	34	6.1	7,.6	1.0
	semi-rigid brace	35	6.1	7.9	

Range of motion (ROM) is defined as the difference between injured and uninjured ankle when substracting the maximal dorsoflexion from plantairflexion. N is number of patients, SD standard deviation.

## Discussion

Functional treatment is a widely used and generally accepted treatment for ankle sprain. A number of studies assessing the effectiveness of different conservative treatments of acute ankle sprain have been performed, but until now, little was known about patient satisfaction in relation to the functional outcome. The results of this randomized controlled trial comparing semi-rigid ankle brace with tape treatment demonstrated improved patient satisfaction with less local complications in patients treated with a semi-rigid brace, but overall showed no improved functional outcome.

Previously, two studies compared patient satisfaction with treatment using brace. In total 76% of patients treated with a brace in the study by Jongen et al. [8] were very satisfied or satisfied with brace treatment, while in our study 95% of patients qualified their satisfaction as excellent or good. This higher percentage may be due to another design of the brace with a more rigid lateral and medial support in our study. Patients in the ankle brace group in the randomized trial from Boyce et al. [16] reported higher levels of comfort and satisfaction, although the used methods to evaluate satisfaction were not specified. The functional outcome Karlsson score was also significantly higher in the brace group compared to that in the elastic bandage group at 10 days and one month.

Kerkhoffs et al. [5] reviewed different functional treatment strategies for acute lateral ankle ligament injuries in adults in a meta-analysis. Although it was impossible to make definitive conclusions about the most effective functional treatment because diversity of outcome results prohibited pooling of results, there seemed to be no evidence that using a semi-rigid brace is superior to taping concerning functional outcome in the individual studies. A semi-rigid ankle support provided more stability and a quicker return to work and sport than an elastic bandage [5]. In addition, as for the functional outcome, objective (ROM) as well as patient-reported functional outcome score (Karlsson scale), this study shows that there was no difference functional ability between the two groups. In addition, the pain score was similar between the tape and semi-rigid brace treatment at 3 months. However, tape treatment resulted in significantly more complications, the majority being skin irritations, when compared with treatment with an elastic bandage [5,8] . In line with these data, this study showed that functional treatment with a semi-rigid brace leads to significant less complications than treatment by taping (RR 0.11; 95% CI 0.01 to 0.86). These results match previous other published studies [5,8-10,16].

A number of remarks must be made when interpreting these observations. Although the loss of follow-up for the primary outcome parameters was 17% at 5 weeks, incomplete data on the secondary outcome parameters was higher with a loss to follow-up for the secondary outcome parameter of 29% at 13 weeks. This loss to follow-up may have introduced misclassification bias [17]. Although the lost-to-follow up was equally distributed among treatment groups and remains below the cut-off value of 80% for the primary outcome parameter, this is not the case for the secondary outcome parameter (Fewtrell MS. Arch Dis Child, 2008;93(6):458-461). Post hoc power analysis indicated that 25 patients should be analyzed in both groups to detect the differences in patients satisfaction score as found in our study. For detecting differences in Karlsson score post hoc power analysis indicated >100.000 patients should be included.

In addition, the costs of treatment with a semi-rigid brace are higher than the treatment with a tape. Diercks et al. [18] described the effectiveness and costs in relation to the patient satisfaction in a small study on the treatment of acute ankle sprain with tape and treatment with a brace and found higher patient satisfaction, but also higher costs of the treatment with a semi-rigid brace (€183 versus €238) Specification of the costs are illustrated in the article by Diercks et al. This comparison seems to be different when tape and brace interventions are used as a preventive measure. In a study by Olmsted et al. found that the costs of preventing one ankle sprain was significantly higher using preventive tape treatment compared to preventive brace treatment [19]. The treatment of an ankle sprain using tape in our study was cheaper mainly due to material costs than treatment with a semi-rigid brace (total costs: €167 (diagnostic costs 121; working costs 27; material costs 8; overhead 11) versus €204, (diagnostic costs 121; working costs 22; material costs 35; overhead 26), respectively). A higher level of comfort during treatment of an ankle sprain therefore comes at the expense at higher treatment costs.

## Conclusion

In summary this study shows that treatment of acute lateral ankle sprain with a semi-rigid brace leads to less complications and a higher patient satisfaction than treatment with tape. In line with previous studies there is no difference regarding functional outcome and pain. Therefore using a semi-rigid brace should be considered for treatment of acute ankle sprains.

## Competing interests

Conflict of interest: none, Bauerfeind provided the materials for the study, but were in no case involved in the setup, data management, and analysis of the study.

## Authors’ contributions

SL and BC initiated the trial, both participated in its design and coordination; SL and ET were responsible for the follow up and data collection; SL and MP had overall responsibility and drafted the manuscript. Statistical analysis and interpretation of data was performed by MP en RAB. PB All authors read and approved the final manuscript.

## Pre-publication history

The pre-publication history for this paper can be accessed here:

http://www.biomedcentral.com/1471-2474/13/81/prepub
